# Serum neurofilament light chain withstands delayed freezing and repeated thawing

**DOI:** 10.1038/s41598-020-77098-8

**Published:** 2020-11-17

**Authors:** Patrick Altmann, Fritz Leutmezer, Heidemarie Zach, Raphael Wurm, Miranda Stattmann, Markus Ponleitner, Axel Petzold, Henrik Zetterberg, Thomas Berger, Paulus Rommer, Gabriel Bsteh

**Affiliations:** 1 Department of Neurology, Medical University of Vienna, Waehringer Guertel 18-20, 1090 Vienna, Austria; 2 Department of Neuroinflammation, UCL Queen Square Institute of Neurology, London, UK; 3 Department of Neurodegenerative Disease, UCL Queen Square Institute of Neurology, London, UK; 4 UK Dementia Research Institute at UCL, London, UK; 5 Department of Psychiatry and Neurochemistry, Institute of Neuroscience and Physiology, the Sahlgrenska Academy at the University of Gothenburg, Mölndal, Sweden; 6 Clinical Neurochemistry Laboratory, Sahlgrenska University Hospital, Mölndal, Sweden

**Keywords:** Biomarkers, Medical research, Neurology

## Abstract

Serum neurofilament light chain (sNfL) and its ability to expose axonal damage in neurologic disorders have solicited a considerable amount of attention in blood biomarker research. Hence, with the proliferation of high-throughput assay technology, there is an imminent need to study the pre-analytical stability of this biomarker. We recruited 20 patients with common neurological diagnoses and 10 controls (i.e. patients without structural neurological disease). We investigated whether a variation in pre-analytical variables (delayed freezing up to 24 h and repeated thawing/freezing for up to three cycles) affects the measured sNfL concentrations using state of the art Simoa technology. Advanced statistical methods were applied to expose any relevant changes in sNfL concentration due to different storing and processing conditions. We found that sNfL concentrations remained stable when samples were frozen within 24 h (mean absolute difference 0.2 pg/ml; intraindividual variation below 0.1%). Repeated thawing and re-freezing up to three times did not change measured sNfL concentration significantly, either (mean absolute difference 0.7 pg/ml; intraindividual variation below 0.2%). We conclude that the soluble sNfL concentration is unaffected at 4–8 °C when samples are frozen within 24 h and single aliquots can be used up to three times. These observations should be considered for planning future studies.

## Introduction

Neurofilaments (Nf) have spurred a compelling field of biomarker research in neurology^1–3^. Several studies underline the capacity of Nf to reliably expose the damage and loss of axons in both cerebrospinal fluid (CSF) and blood for a rising number of various neurologic diseases^4–7^. Historically, one has to name multiple sclerosis (MS) first, where concentrations of serum neurofilament light chains (sNfL) have shaped into a piece in the puzzle that is monitoring of disease and prediction of outcome or therapeutic response^8–10^.

Neurofilaments are part of the axon’s cytoskeleton and, as such, classified as Type IV intermediate filaments consisting of at least four subunits: a light chain (NfL), a medium chain (NfM), a heavy chain (NfH) and alpha-internexin^11,12^. Through the advent of fourth-generation technology, single-molecule array (Simoa)-assays have enabled a precise and more sensitive detection of sNfL in peripheral blood. Starting from a few picograms per milliliter, it is now possible to display a representative range of sNfL concentrations in disease and physiological conditions alike. This at a sensitivity which is able to illustrate and separate the degree of axonal changes that occur in normal ageing, mild head trauma, hypoxia, inflammation or neurodegeneration^10,13–15^.

Nonetheless, in order to successfully launch NfL from bench to bedside, the pre-analytical stability of this biomarker demands careful attention. It is imperative to know about ideal conditions for storage or processing and how even a slight change in these variables may potentially affect measured concentrations. For the analysis of sNfL using Simoa SR-X technology, the consequence of a deviation from standard procedure (i.e. processing blood immediately and freezing at − 70; single use of aliquots) has not been reported to this extent and in three different cohorts^16–18^. Therefore, we studied two important and mutable pre-analytic variables: the effect of delayed freezing (up to 24 h) and repeated thawing and re-freezing (up to three cycles).

## Results

### Patient characteristics

All 30 participants were included in both experimental set ups and the final analysis. Supplementary Table S1 reports on patient characteristics such as age, sex, disease duration, disease phenotype, the clinician reported outcome (CRO) to classify disease severity (Expanded Disability Status Scale [EDSS] for patients with MS and the Hoehn & Yahr scale for patients with Parkinson’s disease [PD]), medication relevant to disease, and baseline sNfL concentrations determined at standard conditions (i.e. processed and frozen immediately and thawed once). Overall, 13 out of 30 included patients were female (43%). Mean age across all three groups was 50.1 years [95% confidence interval (CI) 44.0–56.2]. As for the group of patients with MS, their mean age was 48.6 years (95% CI 43.6–53.6) and median EDSS was 5.0 (range: 1–6.5). Patients with PD had a mean age of 68.9 years (95% CI 63.3–74.5). Median Hoehn & Yahr scale was 2 (range: 1–3). Our controls consisting of patients without structural neurological disease were 32.8 years old (95% CI 27.6–38.0) exhibited non-acute symptoms and were diagnosed with primary headache (n = 5), unspecific dizziness (n = 3) or sinusitis (n = 2). None of the patients included were on any medication affecting blood coagulation or thrombocyte function.

All aliquots of serum samples analyzed in this study yielded a coefficient of variance (CV, i.e. the mean sNfL concentrations as calculated by two replicates) of below 0.2 and internal controls were within the expected range. The mean CV over the whole study was 0.09 (standard deviation: 0.06), suggesting excellent measurement accuracy. No samples were excluded from this analysis.

### sNfL concentrations withstand delayed freezing up to 24 h

**Figure 1:**
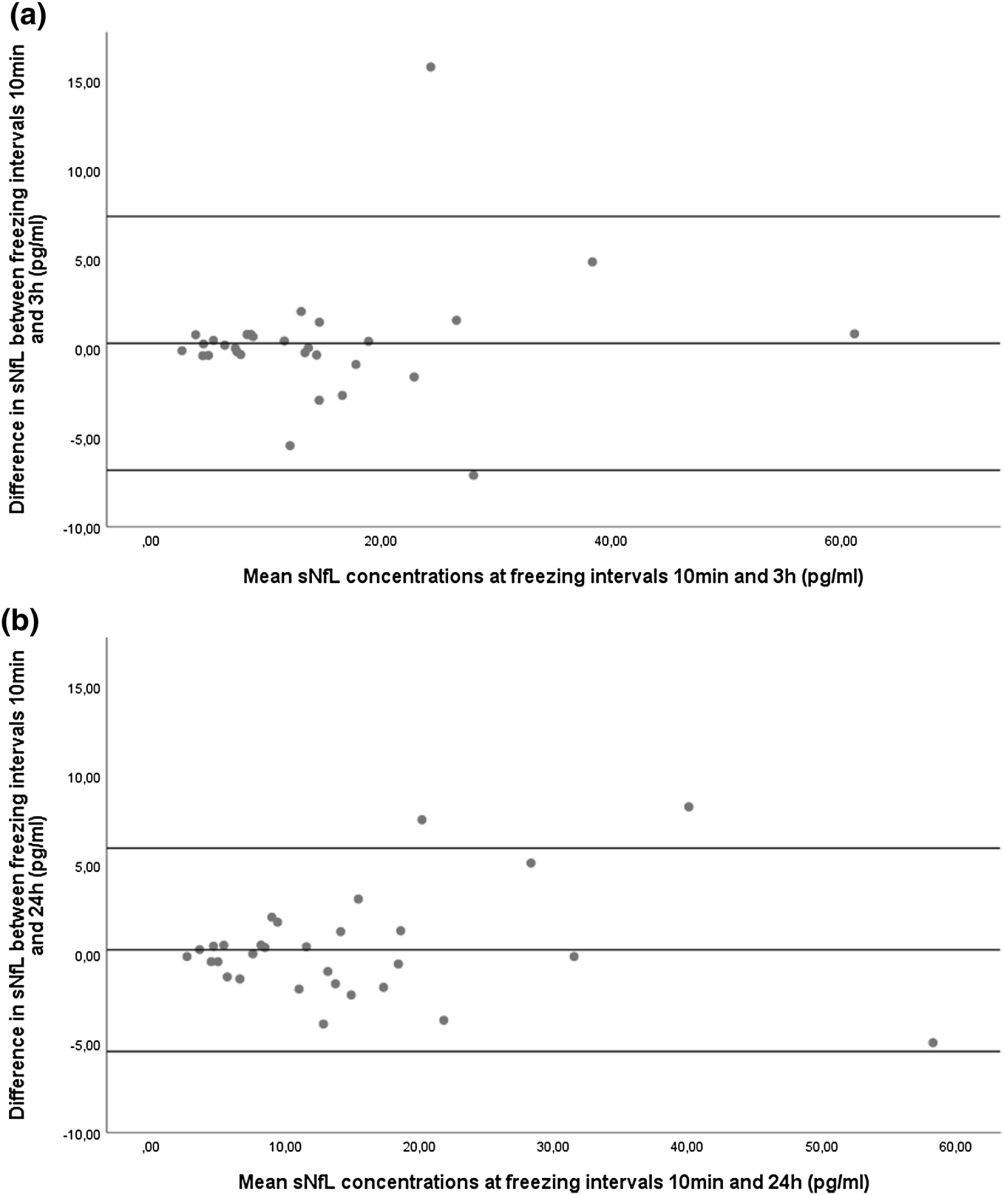
Differences in sNfL concentrations after different freezing intervals. Bland–Altman plot showing the differences in sNfL concentration between two groups of immediate processing and delayed freezing. (**a**) Freezing after 10 min compared to 3 h. (**b**) Freezing after 10 min compared to 24 h. Single dots represent samples at two conditions with their mean concentrations on the x-axis and their difference in concentration on the y-axis. The three horizontal lines represent the mean difference (middle) and the mean difference plus 1.96xSD of that difference (upper) and mean difference minus 1.96xSD of that difference (lower).

Concentrations of sNfL across the three pre-freezing intervals are given in Table 1. For the whole cohort, mean absolute differences in sNfL concentrations compared to immediate freezing were 0.3 pg/ml [intraindividual standard deviations (iSD): 1.28 pg/ml] after three hours and 0.2 pg/ml (iSD: 1.38 pg/ml) after 24 h of delayed freezing (Fig. 1a,b). Reliability of sNfL measurement after different pre-freezing intervals compared to immediate freezing are shown in Table 2. For both pre-freezing intervals, intraindividual variation (iCOV) was minimal (below 0.1%) and reproducibility nearly perfect [intraclass correlation coefficient (ICC) was 0.99]. These findings were similar an all subgroups (MS, PD, controls, lowest quartile, highest quartile).

**Table 1:** Comparison of sNfL concentrations at different freezing intervals.

	[sNfL] freezing interval: 10 min		[sNfL] freezing interval: 3 h		[sNfL] freezing interval: 24 h		*p* value^a^
	Mean	95% CI	Mean	95% CI	Mean	95% CI	
Whole cohort	14.6	10.1–19.1	14.9	10.2–19.6	14.8	10.3–19.4	0.995
MS (n = 10)	11.1	7.4–14.7	11.1	7.5–14.7	10.9	7.4–14.4	0.995
PD (n = 10)	25.7	13.0–37.1	26.3	14.2–31.3	25.8	13.2–38.1	0.973
Controls (n = 10)	8.1	4.6–10.4	8.3	4.7–11.7	8.0	4.6–11.0	0.965
Lowest quartile	4.5	3.5–5.6	4.7	3.5–5.8	4.4	3.5–5.3	0.919
Highest quartile	30.7	17.0–44.3	30.4	15.8–45.0	31.0	17.4–44.5	0.997

CI, confidence interval; sNfL, serum neurofilament light chain concentration [pg/ml]; MS, multiple sclerosis; PD, Parkinson’s disease.

^a^Calculated by repeated measurement ANOVA.

**Table 2:** Reliability of sNfL measurement after different freezing intervals compared to immediate freezing.

	Freezing interval: 3 h			Freezing interval: 24 h		
	iSD (pg/m)	iCOV (%)	ICC	iSD (pg/ml)	iCOV (%)	ICC
Whole cohort	1.28 (0.44–2.11)	0.08 (0.04–0.11)	0.98 (0.95–0.99)	1.38 (0.82–1.95)	0.09 (0.07–0.11)	0.99 (0.97–0.99)
MS (n = 10)	0.29 (0.14–0.43)	0.03 (0.01–0.04)	1.00 (0.99–1.00)	0.67 (0.31–1.03)	0.06 (0.03–0.10)	0.99 (0.95–1.00)
PD (n = 10)	2.89 (0.25–5.56)	0.11 (0.02–0.19)	0.97 (0.87–0.99)	2.62 (1.03–4.19)	0.11 (0.04–0.17)	0.98 (0.91–0.99)
Controls (n = 10)	0.27 (0.08–0.57)	0.09 (0.04–0.15)	0.96 (0.90–0.99)	0.64 (0.36–1.00)	0.10 (0.07–0.15)	0.97 (0.88–0.99)
Lowest quartile	0.26 (0.12–0.40)	0.06 (0.02–0.10)	0.97 (0.83–0.99)	0.35 (0.11–0.59)	0.07 (0.04–0.11)	0.92 (0.54–0.99)
Highest quartile	1.75 (0.09–3.40)	0.06 (0.01–0.12)	0.99 (0.93–1.00)	2.48 (0.58–4.39)	0.08 (0.03–0.13)	0.98 (0.86–1.00)

Mean values with 95% confidence interval. ICC, intraclass correlation coefficient; iCOV, intraindividual coefficient of variance; PD, Parkinson’s disease; iSD, intraindividual standard deviation; MS, multiple sclerosis; sNfL, serum neurofilament light chain concentration (pg/ml).

### sNfL concentrations remain stable in up to three thaw cycles

**Figure 2:**
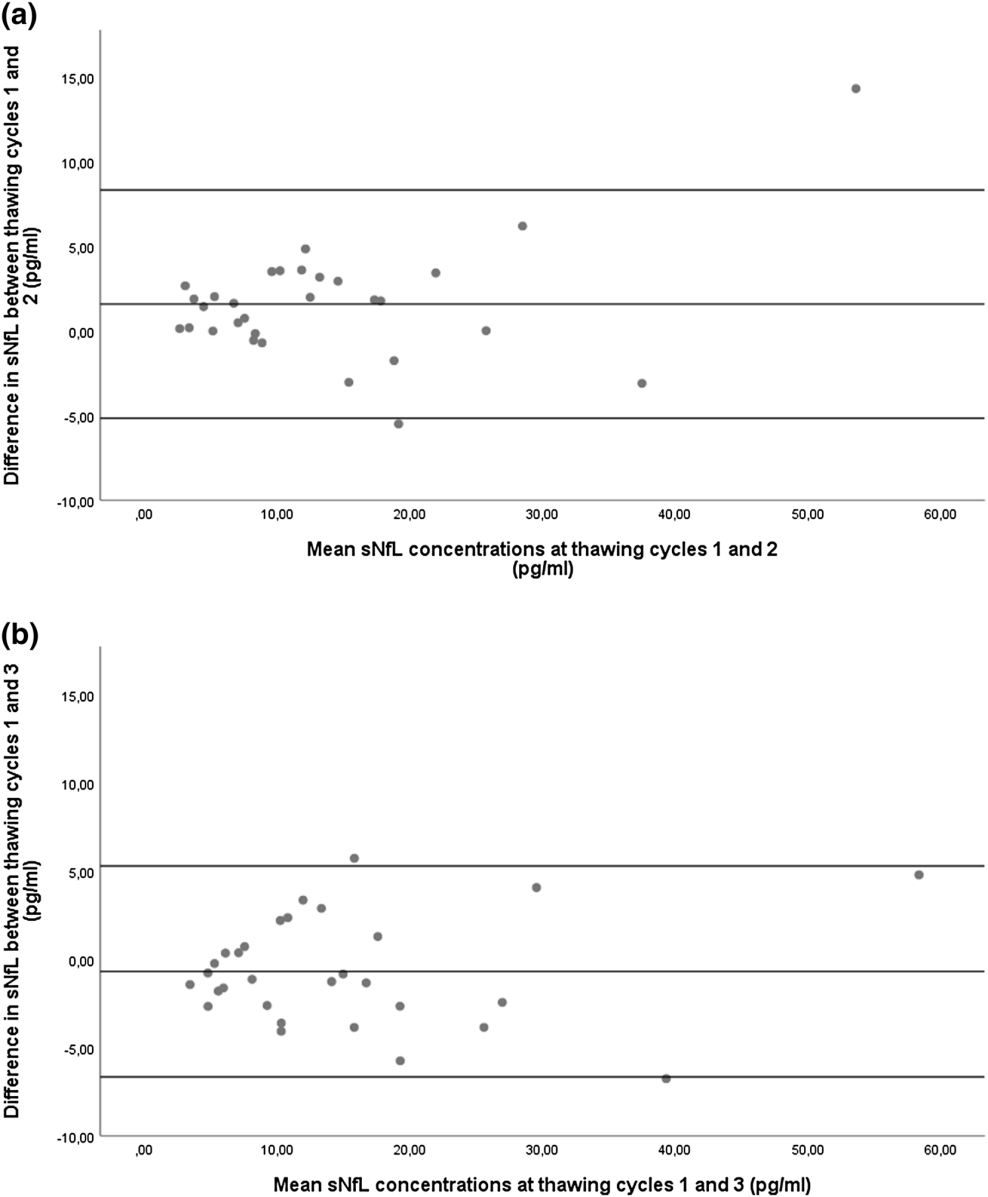
Differences in sNfL concentrations after a different number of thawing cycles. Bland–Altman plot showing the differences in sNfL concentration between two groups of thawing cycles. (**a**) One versus two thawing cycles. (**b**) One vs. three thawing cycles. Single dots represent samples at two conditions with their mean concentrations on the x-axis and their difference in concentration on the y-axis. The three horizontal lines represent the mean difference (middle) and the mean difference plus 1.96xSD of that difference (upper) and mean difference minus 1.96xSD of that difference (lower).

Concentrations of sNfL along three thawing cycles are given in Table 3. For the whole cohort and in comparison to its first thawing cycle, mean absolute differences in sNfL concentrations after a second thawing cycle were 1.6 pg/ml (iSD: 1.8 pg/ml) and 0.7 pg/ml (iSD: 1.8 pg/ml) after a third (Fig. 2a,b). Concerning the reliability of measured sNfL concentration after repeated thawing, intraindividual variation was minimal in the whole cohort (below 0.15%) and reproducibility nearly perfect (ICC = 0.98) (Table 4). These findings were similar in all subgroups (MS, PD, controls, lowest quartile, highest quartile).

**Table 3:** Comparison of sNfL concentrations after three thawing cycles.

	[sNfL] thawing cycle 1		[sNfL] thawing cycle 2		[sNfL] thawing cycle 3		*p* value^a^
	Mean	95% CI	Mean	95% CI	Mean	95% CI	
Whole cohort	14.6	10.1–19.1	13.0	9.1–17.0	15.3	10.9–19.6	0.735
MS (n = 10)	11.1	7.4–14.7	9.5	6.3–12.7	11.3	8.4–14.2	0.643
PD (n = 10)	25.6	15.2–35.9	23.9	15.8–31.9	27.0	17.4–36.6	0.867
Controls (n = 10)	7.2	4.6–9.7	5.7	3.4–7.9	7.5	5.8–9.2	0.380
Lowest quartile	4.5	3.5–5.6	3.4	2.3–4.4	4.7	3.6–5.8	0.732
Highest quartile	30.7	17.0–44.3	27.2	16.6–37.8	30.2	16.6–43.9	0.875

CI, confidence interval; sNfL, serum neurofilament light chain concentration (pg/ml), PD, Parkinson’s disease. ^a^calculated by repeated measurement ANOVA.

**Table 4:** Reliability of sNfL measurement after a second and third thawing cycle compared to one thawing cycle.

	Thawing cycle 2			Thawing cycle 3		
	iSD [pg/ml]	iCOV (%)	ICC	iSD (pg/ml)	iCOV (%)	ICC
Whole cohort	1.8 (1.1–2.5)	0.15 (0.10–0.20)	0.98 (0.95–0.99)	1.8 (1.4–2.3)	0.14 (0.11–0.18)	0.98 (0.96–0.99)
MS (n = 10)	1.2 (0.4–2.0)	0.11 (0.05–0.18)	0.97 (0.87–0.99)	1.6 (0.7–2.4)	0.15 (0.08–0.23)	0.98 (0.89–0.99)
PD (n = 10)	2.9 (0.9–5.0)	0.12 (0.07–0.18)	0.95 (0.77–0.99)	2.5 (1.6–3.5)	0.12 (0.07–0.16)	0.98 (0.91–0.99)
Controls (n = 10)	1.3 (0.7–2.0)	0.11 (0.08–0.14)	0.98 (0.94–0.99)	1.4 (0.8–2.0)	0.12 (0.08–0.15)	0.97 (0.90–0.99)
Lowest quartile	0.8 (0.1–1.5)	0.09 (0.04–0.15)	0.95 (0.73–0.99)	0.9 (0.3–1.5)	0.11 (0.04–0.17)	0.96 (0.92–0.99)
Highest quartile	3.1 (0.1–6.2)	0.22 (0.06–0.31)	0.93 (0.79–0.99)	2.9 (1.7–4.2)	0.19 (0.06–0.31)	0.94 (0.80–0.99)

Mean values with 95% confidence interval. ICC, intraclass correlation coefficient; iCOV, intraindividual coefficient of variance; PD, Parkinson’s disease; iSD, intraindividual standard deviation; MS, multiple sclerosis; sNfL, serum neurofilament light chain concentration [pg/ml].

## Discussion

Serum NfL has turned over a new leaf in biomarker research in neurology. Concurrently, knowledge about the pre-analytical stability of this biomarker should be a prerequisite for establishing study protocols or standard operating procedures. Our study investigated the potential confounder by which measured concentrations of sNfL might change when processing and freezing of serum tubes is delayed up to 24 h in three different cohorts. Furthermore, we analyzed the stability of sNfL after three consecutive cycles of thawing and re-freezing. For both pre-analytical settings, we found that sNfL concentrations remained stable and within acceptable limits. Considered the statistical gold-standard for gauging reproducibility, the ICC takes into account the variance among subjects, the variance among measurements, and the residual error variance. An ICC of 1 represents perfect reproducibility. With an ICC of 0.99 for samples stored at 4–8 °C for 24 h (before being processed and frozen to − 70 °C) and 0.98 for sample aliquots that were thawed and re-frozen three times, we now provide solid evidence that sNfL withstands a delay in freezing for up to 24 h and at least three cycles of repeated thawing and freezing. These results are in line with another investigation looking into the stability of biomarkers in Alzheimer disease over multiple freeze–thaw cycles, including sNfL^16^. For our study, post-hoc power calculations revealed that a systemic measurement error of a mere 2 pg/ml would have been detected at 97% power, further validating the robustness of our results. As importantly, these findings were preserved in samples with different physiological and pathophysiological properties.

Regarding generalizability of our data, there are some limitations. This investigation was performed in a specialized biomarker lab with a protocol specifically evolving around two predetermined hypotheses. Furthermore, our results only apply to the requirements set by our biobanking protocol for handling *serum samples*^19^. It is worth mentioning in this context that plasma samples reportedly yield about 75% of serum NfL^20^. Also, the range of sNfL concentrations we investigated was 2.7–60.8 pg/ml. Therefore, pre-analytical stability may not be assumed for diseases with higher suspected sNfL concentrations. Additionally, the results from this study are derived from only one site with a single person performing sNfL analyses. It does not take into account certain effects or circumstances such as interrater variability or protocol deviations stemming from the busy environment of a routine lab where, usually, various analyses are performed simultaneously and for different analytes. Thus, variance in sNfL levels may be increased in real-world settings. Even so, interrater variability for varying sNfL measurements is considered low and has been reported on already^21^. Besides, our laboratory itself is temperature-controlled, operates at 22–23 °C and does not have any outside-facing windows that could cause disturbances. Interestingly, we noticed a rise in sNfL concentrations over multiple thawing cycles in some cases. While this rise was not statistically significant, it is tempting to speculate that this might be attributed to sample vials being opened and exposed to air more frequently which might cause evaporation resulting in relatively higher protein concentration. This hypothesis might be intriguing to investigate in the future. Ultimately, our study confirms the results of another study published just recently. In this reference sample study, the authors evaluated a subgroup of twelve serum samples for pre-analytical properties and found a stability of 95% for three-day storage at room temperature and 92% after three freeze–thaw cycles compared to standardized conditions^22^.

It is exciting to notice an increase in research invested in the study of intrinsic factors influencing sNfL concentrations in health and disease. A recent cohort study, for example, revealed that healthy individuals exhibit a linear rise in their fourth and fifth decade of life, shifting to a non-linear rise after the sixth. These observations were shown to be related to continuing brain volume loss and were irrespective of sex^23^. Another investigation found a negative correlation between NfL plasma levels and body mass index or blood volume^24^. These are just two examples showing the necessity to understand the multitude of potential influences and confounders in a specific biomarker, in our case, sNfL. This is even more essential as sNfL concentrations (and even more changes in sNfL levels) may occur over the range of some picograms per milliliter.

In summary, we showed that sNfL concentrations remained stable in both our experimental set ups of delayed freezing for up to 24 h and three cycles of repeated thawing and freezing. Therefore, we conclude that serum tubes can be stored up to 24 h in a refrigerator before being processed and thawed/frozen repeatedly for at least three cycles while still yielding reliable results for sNfL.

## Materials and methods

### Ethics and consent

This study was approved by the ethics committee at the Medical University of Vienna (EK2100/2019). Written informed consent was obtained from each patient according to our local biobanking protocol (EK2195/2016). The authors have complied with the World Medical Association Declaration of Helsinki regarding ethical conduct of research involving human subjects.

### Study population

From March 2019 through July 2019, we recruited a total of 30 patients from the outpatient clinic at the Department of Neurology, Medical University of Vienna. They were divided into three predetermined groups: Ten patients diagnosed with MS, ten with PD, and ten controls. These controls were classified as such after their neurologic routine diagnostic workup was unremarkable. This group was comprised of mostly young patients with symptoms such as primary headache or dizziness. Controls’ status was confirmed by two independent reviewers at our department.

### Blood sampling

Peripheral venous blood was collected in Greiner Bio-One Vacuette serum tubes (GBO, Kremsmuenster, Austria) and sent to the local biobank where the blood sample was processed according to standard operating procedures in an ISO 9001-certified environment as described previously^19^. In brief, tubes were centrifuged at 1.884 × *g* for 10 min at room temperature after clotting had completed. Serum was then transferred to 500µL-virgin polypropylene tubes and subsequently stored as 400µL aliquots at − 70 °C (temperature controlled) until analysis.

### Handling of samples for the experimental set up of delayed freezing

To investigate the effect of delayed freezing on sNfL concentrations, three serum tubes from each patient were used. For the first tube, clotting was allowed for 10 min at room temperature, before samples were processed further (i.e. centrifuged and stored in aliquots) in our local biobank. The second tube was placed in a refrigerator (temperature controlled at 4–8 °C) for 3 h before it was sent to the local biobank for processing. The third tube was stored in that same refrigerator for 24 h before being delivered to the biobank.

### Handling of samples for the experimental set up of repeated thawing and re-freezing

The effect of repeated thawing and re-freezing on sNfL levels was studied using aliquots that were handled according to standard procedure (i.e. processed to the biobank without delay). We determined sNfL concentrations three times. For the first thaw cycle, sNfL was measured as described below. After a thawing period of a cumulative three hours, the remaining samples were put back and stored at − 70 °C for two days. For the second thaw cycle, the exact same samples were re-thawed, sNfL concentrations were measured and the remaining samples were again re-frozen at − 70 °C for two days. This process was repeated for the third thaw cycle accordingly.

### Analysis of serum neurofilament light chain concentrations

For sNfL measures, samples were thawed for 60 min at room temperature and were analyzed by an investigator blinded to clinical data using the Simoa Nf-light kits and provided consumables in the Simoa SR-X Analyzer (Quanterix, Lexington, MA, USA)^15^. The sNfL assay was performed according to the manufacturer’s instructions and protocol. Briefly, thawed samples and calibrators were equilibrated to room temperature, diluted in sample diluent (1:4) and dispensed in 96-well plates as duplicates. 20 µl of detector and 25 µl of paramagnetic beads were consecutively dispensed in each well and plates were incubated and shaken (Simoa microplate incubator, 30 °C, 800RPM for 30 min). After pre-set washing steps (Simoa microplate washer), 100 μl streptavidin β-galactosidase was added to each well and plates were incubated (30 °C, 800RPM for 10 min) and washed. After a final washing step, plates were dried for 10 min before being transferred to the Quanterix SR-X analyzer for reading. For the experimental set up of delayed freezing, all samples from one participant were measured on the same plate to avoid inter-assay variability. All assay kits and consumables used in this study were derived from the same kit lot.

### Statistical analyses

Statistical analysis was performed using SPSS 25.0 (SPSS Inc, Chicago, IL, USA). Categorical variables were expressed in frequencies and percentages, continuous variables were tested for normal-distribution by the Shapiro–Wilk test and displayed as mean and 95% CI or median and range as appropriate. Differences between sNfL values after different freezing intervals and thawing cycles were analyzed using repeated measurement ANOVA. A two-sided *p* value of 0.05 was considered the level of significance. Reproducibility of sNfL measurement across all three freezing intervals and thawing cycles was assessed by calculating iSD and iCOV, plotting Bland–Altman plots to analyze the agreement between two conditions for the same sample (these show the mean difference of a sample’s concentration at two conditions on the x-axis and the absolute difference between two samples measured at two conditions on the y-axis) and the applying the formula CV = standard deviation/mean × 100%^25^. In addition, ICC were calculated. ICC calculation is based on a repeated measure ANOVA model using the variance among subjects, the variance among measurements, and the residual error variance^25^. An ICC of 1 represents perfect reproducibility, while an ICC above 0.9 is rated excellent for laboratory tests. ICC was calculated from the sNfL levels after immediate freezing or thawing cycle 1, respectively.

## Supplementary information

Supplementary Table S1.

## Abbreviations

sNfL: Serum neurofilament light chain
Nf: Neurofilament
CSF: Cerebrospinal fluid
MS: Multiple sclerosis
NfL: Neurofilament light chain
NfM: Neurofilament medium chain
NfH: Neurofilament heavy chain
PD: Parkinson’s disease
CI: Confidence interval
iSD: Intraindividual standard deviation
iCOV: Intraindividual coefficient of viariation
ICC: Intraclass correlation coefficient
CRO: Clinician reported outcome
EDSS: Expanded disability status scale
