# Supplementary material for: Serum neurofilament light chain withstands delayed freezing and repeated thawing
Source: Sci Rep. 2020 Nov 17;10:19982. doi: 10.1038/s41598-020-77098-8 (PMC7672085; doi:10.1038/s41598-020-77098-8)
Supplement: Supplementary file 1 — Supplementary Table S1. [file 41598_2020_77098_MOESM1_ESM.docx]

**Supplementary table S1 as part of the submission:**

**Serum neurofilament light chain withstands delayed freezing and repeated thawing**

Patrick Altmann^1^, Fritz Leutmezer^1^, Heidemarie Zach^1^, Raphael Wurm^1^, Miranda Stattmann^1^, Markus Ponleitner^1^, Axel Petzold^2^, Henrik Zetterberg^3,4,5,6^, Thomas Berger^1^, Paulus Rommer^1^, Gabriel Bsteh^1^

^1^ Medical University of Vienna, Department of Neurology, Vienna, Austria

^2^ UCL Queen Square Institute of Neurology, Department of Neuroinflammation, London, UK

^3^ UCL Queen Square Institute of Neurology, Department of Neurodegenerative Disease, London, UK

^4^ UK Dementia Research Institute at UCL, London, UK

^5^ Department of Psychiatry and Neurochemistry, Institute of Neuroscience and Physiology, the Sahlgrenska Academy at the University of Gothenburg, Mölndal, Sweden

^6^ Clinical Neurochemistry Laboratory, Sahlgrenska University Hospital, Mölndal, Sweden

**Revised version 1.1**

**Corresponding Author:**

Gabriel Bsteh, MD PhD

Waehringer Guertel 18-20

A‑1090 Vienna, Austria

*Phone:* +43-1-40400-31450

*Fax:* +43-1-40400-31410

[gabriel.bsteh@meduniwien.ac.at](mailto:gabriel.bsteh@meduniwien.ac.at)

**Supplementary Table S1: Patient characteristics**

| **Patient ID** | **Diagnosis** | **Age** | **Sex** | **Disease duration (years)** | **CRO** | **Phenotype/diagnosis** | **Disease-specific medication** | sNfL [pg/ml] |
| --- | --- | --- | --- | --- | --- | --- | --- | --- |
| 1 | MS | 49 | f | 18 | 2 | RRMS | Intravenous Immunoglobulin | 11.3 |
| 2 | MS | 44 | m | 5 | 4.5 | SPMS | Fingolimod | 7.3 |
| 3 | MS | 31 | m | 8 | 6.5 | SPMS | Rituximab | 8.5 |
| 4 | MS | 55 | f | 22 | 6.5 | SPMS | none | 18.2 |
| 5 | MS | 55 | m | 16 | 6 | SPMS | Rituximab | 13.5 |
| 6 | MS | 53 | m | 22 | 6.5 | SPMS | none | 14.6 |
| 7 | MS | 48 | f | 16 | 3.5 | RRMS | Fingolimod | 7.5 |
| 8 | MS | 46 | m | 3 | 6.5 | RRMS | none | 18.7 |
| 9 | MS | 43 | m | 7 | 4 | RRMS | none | 2.7 |
| 10 | MS | 62 | f | 23 | 4 | SPMS | Interferon beta | 8.3 |
| 11 | PD | 82 | m | 4 | 2 | PD | L-DOPA | 60.8 |
| 12 | PD | 77 | f | 8 | 2.5 | PD | L-DOPA, MAO, DA | 35.9 |
| 13 | PD | 53 | f | 0.1 | 2 | PD | DA | 16.4 |
| 14 | PD | 69 | m | 10 | 1.5 | PD | L-DOPA, MAO | 31.6 |
| 15 | PD | 76 | m | 0.2 | 1 | PD | L-DOPA | 25.7 |
| 16 | PD | 73 | f | 8 | 1.5 | PD | L-DOPA, MAO, DA | 17.9 |
| 17 | PD | 61 | f | 7 | 1 | PD | L-DOPA, MAO, AMA | 16.0 |
| 18 | PD | 57 | m | 7 | 1 | PD | L-DOPA, MAO, DA | 13.6 |
| 19 | PD | 65 | f | 5 | 3 | PD | L-DOPA, DA | 13.9 |
| 20 | PD | 76 | f | 12 | 1 | PD | L-DOPA, DA | 23.7 |
| 21 | Control | 26 | f | / | / | Primary headache | / | 7.9 |
| 22 | Control | 38 | m | / | / | Sinusitis | / | 12.0 |
| 23 | Control | 26 | m | / | / | Sinusitis | / | 4.4 |
| 24 | Control | 35 | m | / | / | Dizziness | / | 5.2 |
| 25 | Control | 26 | m | / | / | Primary headache | / | 6.3 |
| 26 | Control | 25 | f | / | / | Dizziness | / | 4.7 |
| 27 | Control | 48 | m | / | / | Primary headache | / | 14.8 |
| 28 | Control | 22 | m | / | / | Dizziness | / | 3.5 |
| 29 | Control | 41 | f | / | / | Primary headache | / | 7.9 |
| 30 | Control | 41 | m | / | / | Primary headache | / | 5.1 |

Supplementary Table S1: Patient characteristics.

AMA, amantadine; CRO, clinician reported outcomes (numbers indicating scores on the Expanded Disability Status Scale for patients with MS and the Hoehn & Yahr scale for patients with PD); DA, dopamine agonist; f, female; L-DOPA, Levodopa; m, male; MAO, monoamine oxidase inhibitor; MS, Multiple Sclerosis; PD, Parkinson’s disease; RRMS, relapsing remitting MS; sNfL, serum neurofilament light chain; SPMS, secondary progressive MS.
